# The Three-Month Effects of a Ketogenic Diet on Body Composition, Blood Parameters, and Performance Metrics in CrossFit Trainees: A Pilot Study

**DOI:** 10.3390/sports6010001

**Published:** 2018-01-09

**Authors:** Wesley C. Kephart, Coree D. Pledge, Paul A. Roberson, Petey W. Mumford, Matthew A. Romero, Christopher B. Mobley, Jeffrey S. Martin, Kaelin C. Young, Ryan P. Lowery, Jacob M. Wilson, Kevin W. Huggins, Michael D. Roberts

**Affiliations:** 1 School of Kinesiology, Auburn University, Auburn, AL 36849, USA; kephartw@uww.edu (W.C.K.); par0021@auburn.edu (P.A.R.); pwm0009@auburn.edu (P.W.M.); mzr0049@auburn.edu (M.A.R.); moblecb@auburn.edu (C.B.M.); jmartin@auburn.vcom.edu (J.S.M.); kyoung@auburn.vcom.edu (K.C.Y.); 2 Department of Health, Physical Education, Recreation and Coaching, University of Wisconsin-Whitewater, Whitewater, WI 53190, USA; 3 Department of Nutrition, Dietetics and Hospitality Management, Auburn University, Auburn, AL 36849, USA; cdp0017@auburn.edu (C.D.P.); huggikw@auburn.edu (K.W.H.); 4 Department of Cell Biology and Physiology, Edward Via College of Osteopathic Medicine, Auburn, AL 36830, USA; 5 Applied Sports Performance Institute, Tampa, FL 33607, USA; rlowery@theaspi.com (R.P.L.); jwilson@theaspi.com (J.M.W.)

**Keywords:** ketogenic diet, body composition, power, strength

## Abstract

Adopting low carbohydrate, ketogenic diets remains a controversial issue for individuals who resistance train given that this form of dieting has been speculated to reduce skeletal muscle glycogen levels and stifle muscle anabolism. We sought to characterize the effects of a 12-week ketogenic diet (KD) on body composition, metabolic, and performance parameters in participants who trained recreationally at a local CrossFit facility. Twelve participants (nine males and three females, 31 ± 2 years of age, 80.3 ± 5.1 kg body mass, 22.9 ± 2.3% body fat, 1.37 back squat: body mass ratio) were divided into a control group (CTL; *n* = 5) and a KD group (*n* = 7). KD participants were given dietary guidelines to follow over 12 weeks while CTL participants were instructed to continue their normal diet throughout the study, and all participants continued their CrossFit training routine for 12 weeks. Pre, 2.5-week, and 12-week anaerobic performance tests were conducted, and pre- and 12-week tests were performed for body composition using dual X-ray absorptiometry (DXA) and ultrasound, resting energy expenditure (REE), blood-serum health markers, and aerobic capacity. Additionally, blood beta hydroxybutyrate (BHB) levels were measured weekly. Blood BHB levels were 2.8- to 9.5-fold higher in KD versus CTL throughout confirming a state of nutritional ketosis. DXA fat mass decreased by 12.4% in KD (*p* = 0.053). DXA total lean body mass changes were not different between groups, although DXA dual-leg lean mass decreased in the KD group by 1.4% (*p* = 0.068), and vastus lateralis thickness values decreased in the KD group by ~8% (*p* = 0.065). Changes in fasting glucose, HDL cholesterol, and triglycerides were similar between groups, although LDL cholesterol increased ~35% in KD (*p* = 0.048). Between-group changes in REE, one-repetition maximum (1-RM) back squat, 400 m run times, and VO_2peak_ were similar between groups. While our *n*-sizes were limited, these preliminary data suggest that adopting a ketogenic diet causes marked reductions in whole-body adiposity while not impacting performance measures in recreationally-trained CrossFit trainees. Whether decrements in dual-leg muscle mass and vastus lateralis thickness in KD participants were due to fluid shifts remain unresolved, and increased LDL-C in these individuals warrants further investigation.

## 1. Introduction

Dietary practices that facilitate losses in body fat while maintaining or leading to an accretion of muscle mass have been of interest to individuals who resistance train recreationally. In this regard, there is overwhelming support that higher protein diets are able to improve muscle mass gains while not affecting body fat [1,2,3,4]. However, more recent data from our laboratory in rodents [5,6], and other evidence in humans [7], suggest that adopting a low-carbohydrate, moderate-protein, high-fat ketogenic diet (KD) may also serve to support muscle mass gains with resistance training while promoting decreases in adiposity. Notwithstanding, there are still scientific and mainstream commentaries that posit this form of dieting is detrimental to individuals who resistance train. Namely, given that the diet is very low in carbohydrates, it has been posited that a KD will reduce skeletal muscle glycogen levels and chronically increase intramuscular AMP-activated protein kinase (AMPK) signaling. Indeed, these findings have been reported in mice [8,9], and have led the authors to speculate that KD feeding may lead to a chronic suppression of mammalian target of rapamycin complex 1 (mTORc1) signaling and muscle protein synthesis, as well as an eventual stagnation in the anabolic response to resistance training and/or a loss in muscle mass in humans.

To counter these hypotheses, we have demonstrated that six weeks of KD feeding in rats does not impair muscle glycogen levels, reduce mTORc1 signaling markers, or affect basal muscle protein synthesis levels when compared with rats fed an isocaloric Western diet (WD) [5]. Additionally, we observed that KD- and Western diet (WD)-fed rats experienced similar increases in hind limb muscle masses and ribosome biogenesis when voluntarily trained on resistance-loaded running wheels over a six-week period. In humans, Paoli et al. [10] reported that elite male artistic gymnasts adopting a KD for 30 days during a normal training schedule experienced a robust decrease in whole-body adiposity, but no significant impairment of muscular strength or reduction in muscle mass. Wilson et al. [7] recently reported that male bodybuilders adopting a KD for 10 weeks experienced similar increases in muscle mass and strength, as well as similar reductions in fat mass, compared with bodybuilders consuming a mixed macronutrient diet. Gregory et al. [11] reported that six weeks of KD reduced fat mass by 2.8 kg on average without affecting lean body mass in non-elite CrossFit athletes. Additionally, power metrics, along with time to complete a circuit-training style workout, was not impaired when compared with athletes consuming their typical diet. Hence, these recent data counter molecular evidence indicating that adopting a KD will lead to muscle mass loss or performance decrements in anaerobic athletes. It should be noted, however, that recent data also indicate that shorter-term ketogenic diets could negatively alter physiological adaptations to aerobic training. In this regard, Burke et al. [12] reported that a three-week KD increased the oxygen cost during a two-hour treadmill race-walk in world-class race walkers. Thus, more studies examining the physiological and performance effects of ketogenic diets are warranted.

Herein, we sought to characterize the effects of a 12-week KD on body composition, metabolic, and performance parameters in participants who trained recreationally at a local CrossFit facility. Based on the supporting literature, we hypothesized that participants would experience reductions in whole-body adiposity but would not experience losses in lean body mass or impairments in performance metrics.

## 2. Materials and Methods

### 2.1. Ethics Approval and Subject Recruitment and Consent

Prior to initiating this study, the protocol was reviewed and approved by the Auburn University Institutional Review Board (approved protocol #: 16-031 MR 1603; IRB contact: irbadmin@auburn.edu) and was in compliance with the Helsinki Declaration. Twelve subjects were recruited from a local CrossFit gymnasium in the Auburn community to participate in the study. As inclusion criteria, we established that participants must have: (a) been active members at the gymnasium for at least three months; (b) been between the ages of 19–45; and (c) displayed a strength:mass ratio (determined via one-repetition maximum back squat) of at least 1.00.

### 2.2. Study Design

**Figure 1:**
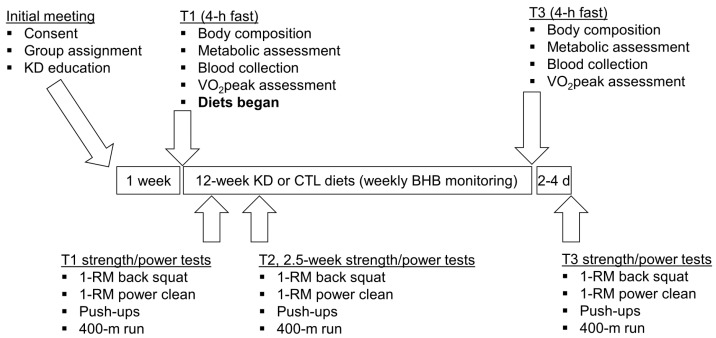
Study design. This figure depicts the 12-week dietary intervention. Notably, all participants continued their normal workout routine at the local CrossFit training facility during the study. Abbreviations: KD, ketogenic diet; CTL, control diet; 1-RM, one repetition maximum.

A depiction of the study design is presented in Figure 1. Briefly, an initial meeting at the School of Kinesiology at Auburn University between study investigators and participants was held whereby all 12 participants were informed about the purpose of the study and the study design, and educated about the KD. Thereafter, participants were given a choice to participate in the KD group or continue their normal diet (CTL). In an effort to optimize compliance with KD guidelines, participants who verbalized interest with adopting a KD for 12 weeks were placed in the KD group (*n* = 7), while others were placed in the CTL group (*n* = 5). Participants then completed a screening and health history questionnaire to ensure inclusion criteria were met and that there were no risk factors that might be aggravated by maximal free-weight strength testing and aerobic testing (e.g., musculoskeletal injuries or exercise-induced asthma). Participants were then told to maintain their pre-study diet and scheduled for baseline testing which commenced ~1 week following the initial meeting.

### 2.3. Body Composition, Metabolic, and Aerobic Performance Assessments

During T1 (pre-test) and T3 (post-test) participants were instructed to arrive to the School of Kinesiology at Auburn University either in the morning or evening in a 4-h fasted state in order to best fit their work schedules; notably T1 and T3 time of day testing was similar for each participant. Upon arrival to the laboratory, participants submitted a urine sample (~5 mL) to assess for normal hydration specific gravity levels (1.005–1.020 ppm) using a handheld refractometer (ATAGO; Bellevue, WA, USA). Participants with a urine specific gravity ≥1.020 were asked to consume tap water every 15 min for 30 min and then were re-tested. Following hydration testing, height (T1 only) and body mass were assessed using a digital column scale (Seca 769; Seca, Chino, CA, USA). Next, participants were subjected to a full body dual X-ray absorptiometry (DXA) scan while wearing general sports attire (i.e., athletic shorts or compression shorts and an athletic shirt) to assess various body composition characteristics. Notably, body segmentation for each scan was standardized prior to analyses by the same technician (Wesley C. Kephart), and total body lean mass and fat mass was assessed by the accompanying software. According to previous data published by our laboratory, the same-day reliability of the DXA during a test-calibrate-retest on 10 participants produced intra-class correlation coefficients of 0.998 for total body fat mass (mean difference between tests = 0.40 ± 0.05 kg), and 0.998 for total lean body mass (mean difference between tests = 0.29 ± 0.13 kg) [13].

Following body composition assessments, resting energy expenditure (REE) and respiratory quotient (RQ) assessments were performed in a supine position over a 10-min period in a dark and quite laboratory space. Expired gases during the tests were continuously analyzed over the 10-min period using a TrueMax 2400 metabolic measurement system (ParvoMedics, Sandy, UT, USA) with a dilution pump which was used to set the fraction of end tidal CO_2_ (FECO_2_) between 0.90–1.10. All data were averaged in 1 min intervals, and the average of the last 5 min of the test was used for REE and RQ calculations.

Following REE assessments, participants donated a venous blood sample (described in greater detail below), and then performed VO_2peak_ testing on a motorized treadmill (Waukesha, Woodway, WI, USA) using a graded protocol. Briefly, participants were allowed to warm-up by walking on the treadmill at 4.8 km/h (3.0 mph) for 3 min. After the warm-up was completed, the treadmill incline was set to 1% and belt speed was set to the speed pre-indicated by the participants as a comfortable 5-km running pace. Thereafter, the treadmill grade increased by 2% every 2 min until the participants indicated volitional fatigue. Expired gases during the tests were continuously analyzed using a TrueMax 2400 metabolic measurement system (ParvoMedics, Sandy, UT, USA), averaged in 15-s intervals, and the highest 15-s average for VO_2_ was denoted as the VO_2peak_.

### 2.4. Blood-Serum Collection and Analysis

Blood was collected in a 4-h fasted state at T1 and T3 from the antecubital vein and allowed to clot at room temperature. Thereafter, tubes were centrifuged at 3500 *g* (23 °C) for 10 min. Serum was aliquoted in 1.7 mL microtubes and stored at −80 °C. Once the study was completed, all tubes were transported to the East Alabama Medical Center (Opelika, AL, USA) whereby blood glucose and lipids were determined. Additionally, whole-blood (2-h post prandial) beta-hydroxbutyrate (BHB) concentrations were determined weekly via finger sticks using a handheld analyzer and testing strips (CardioChek, Indianapolis, IN, USA).

### 2.5. Anaerobic Performance Assessments

Approximately 48–96 h following T1 and T3, as well as two and one-half weeks into the intervention, participants performed one repetition maximum (1-RM) back squat and power clean assessments using a 20 kg barbell (York Barbell) and free weights at the local CrossFit gymnasium (Backbone CrossFit, Auburn, AL, USA). The demonstration of proper technique as well as the implementation of progressively-loaded RM testing were overseen by Wesley C. Kephart and Coree D. Pledge, who have vast experience with strength and performance testing [14,15,16]. A repetition was not counted if subjects exhibited poor and/or unsafe technique or needed assistance with a repetition during maximal testing. Approximately 10 min following 1-RM assessments, maximal repetition pushup tests were performed to volitional fatigue. Finally, 400-m run assessments were performed on an outdoor recreational track (400 m/lap) using a handheld timer. Unlike the laboratory tests, where participants were instructed to arrive following a 4-h fast, no mandated fasting was implemented prior to these performance tests.

### 2.6. Dietary Guidelines and Nutritional Intake Monitoring

Following the T1 anaerobic testing procedures, participants in the KD group were given general KD guidelines and handouts of sample meal plans published by Volek and Phinney [17]. Participants in the CTL group were instructed to maintain their normal dietary habits throughout the study. Only KD subjects were asked to complete three-day food logs (two weekdays and one weekend day) to return within one week of T1 and T3 testing. On each occasion, participants were given detailed written and verbal instructions on completing the food logs. Dietary intake data were analyzed using open-sourced software (http://www.myfitnesspal.com), which has been employed by our laboratory to analyze macronutrient data [15].

### 2.7. Statistical Analyses and Data Presentation

All data herein are presented in figures as means ± standard error values with individual subject plots and statistical analyses were performed using SPSS v22.0 (IBM, Armonk, NY, USA). With the exception of weekly blood BHB levels, which were compared between groups using independent samples *t*-tests, all dependent variables were examined between groups using two-way (group × time) repeated measures analysis of variances (ANOVAs). Huynh-Feldt (H-F) corrections were applied for data where the assumption of sphericity was violated. Given our limited *n*-sizes, partial eta-squared values (η_p_^2^) for group × time interactions, and interactions were further interrogated if they were significant (*p* < 0.05) or effect sizes were large (η_p_^2^ > 0.14). In these cases, within-group dependent samples *t*-tests as well as between-group independent samples *t*-tests were performed as post hoc tests, and *p*-values between 0.05 and 0.10 (i.e., ‘approaching significance’) were considered meaningful due to limited *n*-sizes.

## 3. Results

### 3.1. Baseline Characteristics between Treatments

Baseline characteristics between groups are presented in Table 1. Independent *t*-tests indicated that there were no between-group differences for age, height, body mass, DXA body fat percentage, or strength:mass ratio. Notably, one KD participant presented with a strength: body mass ratio of 0.93, but the investigators decided to include the subject in the analyses due to limited *n*-sizes.

### 3.2. Self-Reported Macronutrient Intakes and Workouts Completed over the Intervention

As noted previously, only the KD participants completed food logs. Of the seven KD participants, only four returned the T3 food logs. Notably, self-reported carbohydrate consumption decreased (T1 = 164 ± 32 g/d, T3 = 15 ± 3 g/d, *p* = 0.014) and total calorie consumption decreased (T1 = 2499 ± 350 kcal/d, T3 = 1948 ± 293 kcal/d, *p* = 0.032). While self-reported dietary fat values were greater (T1 = 154 ± 40 g/d, T3 = 170 ± 25 g/d, *p* = 0.576) and protein values were lower (T1 = 114 ± 10 g/d, T3 = 89 ± 20 kcal/d, *p* = 0.112), there were no significant differences between pre- and post-values. Additionally, while three participants failed to return post-study food logs, weeks 1–12 average blood ketone levels for these participants were 0.87, 0.95, and 1.43 μM, suggesting that they complied with the KD guidelines throughout the study.

Regarding CrossFit workouts completed over the intervention, the KD group completed an average of 27 ± 3 workouts, whereas the CTL group completed an average of 20 ± 5 workouts (*p* = 0.245 between conditions). 

### 3.3. Weekly Blood Ketone Levels and Changes in Body Composition Metrics

**Figure 2:**
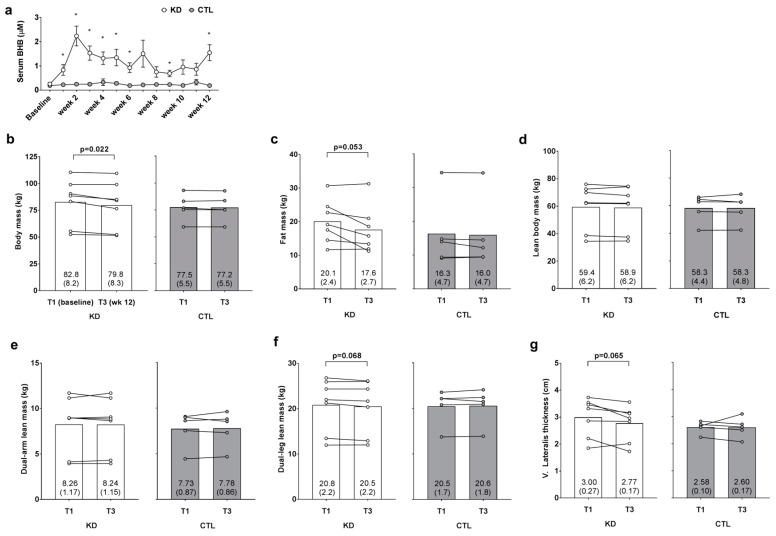
Weekly blood ketone and body composition effects prior to and 12 weeks following the study. Weekly blood beta-hydroxybutyrate (BHB) levels are presented in panel (**a**); DXA body mass, DXA fat mass, DXA lean body mass, DXA dual-arm lean mass, DXA dual-leg lean mass, and vastus lateralis thickness (assessed via ultrasound) are presented in panels (**b**–**g**); Data in panel (**a**) are presented as means ±SE; Bar graph data in panels (**b**–**g**) are presented as group means accompanied by individual participant values, and means (±SE) values are indicated at the bottom portion of each bar. Abbreviations: KD, ketogenic diet; CTL, control diet; T1, baseline testing; T3, 12-week post-testing. Symbols: * indicates between-group difference in blood BHB (*p* < 0.05).

Blood BHB levels were 2.8–9.5-fold higher in KD versus CTL throughout the intervention confirming a state of nutritional ketosis in the KD group. In addition, independent samples *t*-tests indicated that humoral BHB levels were significantly greater (*p* < 0.05) in the KD group at weeks 1–6, 9 and 12 (Figure 2a). A main time effect (T1 > T3; *p* = 0.020) and large effect for the group × time interaction was observed for change in body mass between groups (*p* = 0.053, η_p_^2^ = 0.325; Figure 2b); notably a significant decrease from T1 within the KD group was observed (*p* = 0.022). Additionally, a main time effect approached significance (T1 > T3; *p* = 0.051), and a large effect for the group × time interaction was observed for change in DXA fat mass between groups (*p* = 0.126, η_p_^2^ = 0.218; Figure 2c); notably, the decrease from T1 within the KD group approached significance (*p* = 0.053). There was no main time effect (*p* = 0.521) or group × time interaction observed for DXA lean body mass (*p* = 0.534, η_p_^2^ = 0.040; Figure 2d). There was no main time effect (*p* = 0.876) or group × time interaction observed dual-arm DXA lean mass (*p* = 0.742, η_p_^2^ = 0.011; Figure 2e). There was no main time effect for dual-leg DXA lean mass (*p* = 0.344), although a large effect for the group × time interaction was observed (*p* = 0.111, η_p_^2^ = 0.234; Figure 2f) whereby lower T3 values in the KD group approached significance (*p* = 0.068). Likewise, there was no main time effect for ultrasound derived vastus lateralis thickness (*p* = 0.159), although a large effect for the group × time interaction was observed (*p* = 0.180, η_p_^2^ = 0.172; Figure 2e) whereby lower T3 values in the KD group approached significance (*p* = 0.065).

### 3.4. Changes in Resting Metabolic Metrics

**Figure 3:**
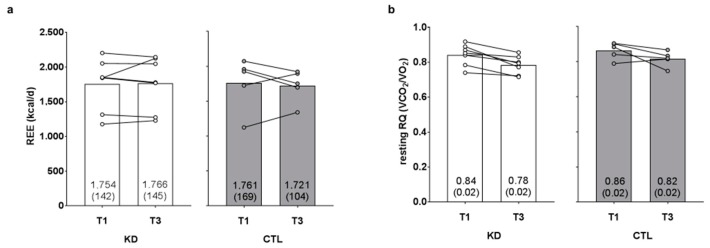
Effects of the intervention on resting energy expenditure and respiratory quotient values. Resting energy expenditure (REE) and resting respiratory quotient (RQ) values are presented in panels (**a**,**b**). Data are presented as group means accompanied by individual participant values, and means (±SE) values are indicated at the bottom portion of each bar. Abbreviations: KD, ketogenic diet; CTL, control diet; T1, baseline testing; T3, 12-week post-testing.

There was no time (*p* = 0.781) or group × time interaction observed for resting energy expenditure (*p* = 0.613, η_p_^2^ = 0.024; Figure 3a). There was a significant time effect (T1 > T3, *p* = 0.002) observed for resting RQ, although there was no group × time interaction (*p* = 0.916, η_p_^2^ = 0.001; Figure 3b).

### 3.5. Changes in Performance Metrics

**Figure 4:**
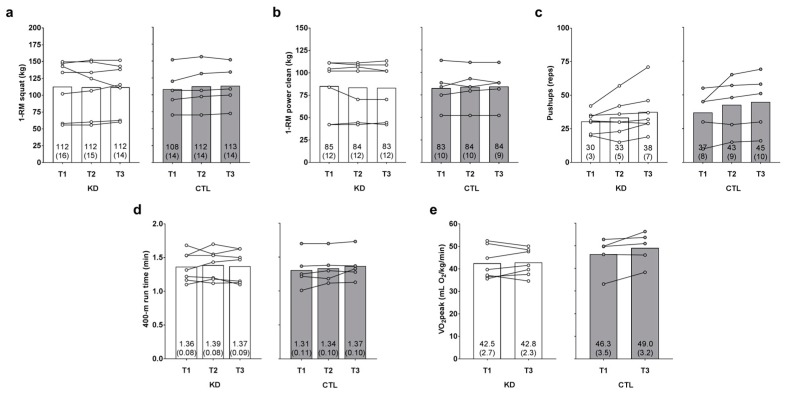
Effects of the intervention on strength, anaerobic, and aerobic performance metrics. One-repetition maximum (1-RM) squat, 1-RM power clean, maximum pushup repetitions, 400-m run time, and VO_2peak_ values (assessed with a graded treadmill test and indirect calorimetry) are presented in panels (**a**–**e**). Data are presented as group means accompanied by individual participant values, and means (±SE) values are indicated at the bottom portion of each bar. Abbreviations: KD, ketogenic diet; CTL, control diet; T1, baseline testing; T2, 2.5-week testing; T3, 12-week post-testing.

There was no main time effect [Huynh-Feldt (H-F) *p* = 0.626] or group × time interaction observed for 1-RM squat (H-F *p* = 0.422, η_p_^2^ = 0.073; Figure 4a). There was no main time effect (*p* = 0.995) or group × time interaction observed for 1-RM power clean (*p* = 0.347, η_p_^2^ = 0.100; Figure 4b).

There was a main time effect regarding maximum pushup repetitions (T3 > T1 and T2, *p* = 0.032 and *p* = 0.016, respectively), but no group × time interaction was observed (H-F *p* = 0.716, η_p_^2^ = 0.020; Figure 4c). There was no main time effect (*p* = 0.326) or group × time interaction observed for 400-m run time (*p* = 0.479, η_p_^2^ = 0.071; Figure 4d). There was no main time effect observed for observed for VO_2peak_ (*p* = 0.105), although a large effect for the group × time interaction was observed (*p* = 0.188, η_p_^2^ = 0.166; Figure 4e). However, numerically greater T3 values in the KD (*p* = 0.782) and CTL (*p* = 0.109) groups did not approach statistical significance.

### 3.6. Changes in Blood-Serum Parameters

Changes in blood parameters between groups are presented in Table 2. There was no main time effect (*p* = 0.190) or group × time interaction observed for serum glucose (*p* = 0.881, η_p_^2^ = 0.002). There was no main time effect (*p* = 0.409) or group × time interaction observed for HDL cholesterol (*p* = 0.649, η_p_^2^ = 0.022). There was no main time effect (*p* = 0.977) or group × time interaction observed for triglycerides (*p* = 0.585, η_p_^2^ = 0.031). There was no main time effect (*p* = 0.147), although there was a significant and large effect for the group × time interaction observed for serum LDL cholesterol levels (*p* = 0.029, η_p_^2^ = 0.393), and post hoc tests indicated within-group increases for the KD group (*p* = 0.048) and significant decreases in the CTL group (*p* = 0.006).

## 4. Discussion

This is one of the first reports to examine the longer-term physiological and performance effects of a ketogenic diet in recreationally resistance-trained individuals. As mentioned previously, we have demonstrated in rats that six weeks of KD feeding does not adversely affect markers of muscle hypertrophy, and that KD- and WD-fed rats experienced similar increases in hind limb muscle masses and molecular markers suggestive of anabolism when voluntarily trained on resistance-loaded running wheels over a six-week period [5]. Likewise, Paoli et al. [10], Wilson et al. [7] and Gregory et al. [11] reported that humans engaging in a KD do not experience anaerobic performance deficits over a 4–10-week period. Herein, we add to these data to suggest that individuals who train recreationally at a CrossFit gym while adopting a KD for 12 weeks experience a reduction in whole-body adiposity with little influence on metabolic or exercise performance measures. It is notable, however, that KD participants did experience decrements in vastus lateralis thickness and dual-leg DXA lean mass following the intervention. These findings counter the Paoli et al. [10], Wilson et al. [7], and Gregory et al. [11] data, although several studies have reported that engaging in a 12-week KD decreases DXA lean body mass in individuals who participated in a diet-only intervention [18,19,20]. Notably, our body composition findings could be indicative of resultant fluid shifts that occur via KD. While we did confirm a state of adequate hydration via urinalysis, others have reported that four months of KD elicited a marked reduction in DXA lean body mass primarily due to changes in total body water (assessed via bioelectrical impedance) [21]. Given these discordant data, more human studies are needed for clarity regarding shorter-term and longer-term KD effects on whole-body hydration, protein metabolism, muscle protein synthesis levels, and muscle fiber histological attributes (i.e., muscle fiber cross sectional area) in exercise-trained individuals.

While no performance decrements were evident in KD participants following the intervention, it is notable that these participants did not experience improvements in certain performance measures relative to CTL participants (e.g., 1-RM squat and VO_2peak_). Alternatively stated, the CTL group may have experienced significant improvements in select performance measures had *n*-sizes been larger or the study had been longer in duration. Select commentaries have asserted that individuals who aim to optimize anaerobic endeavors benefit from higher amounts of dietary carbohydrates; case in point, a recent commentary suggests that consuming 2.5 g/kg 3 h prior to exercise and 9 g/kg during post-exercise recovery will benefit stop-and-go athletes [22]. While individuals in the current study were recreationally trained, we observed no performance decrements 2.5 weeks or 12 weeks after engaging in a KD. Indeed, our data agree with others who reported that KD does not adversely affect anaerobic performance [7,10]. Moreover, others have reported that aerobic-based athletes experience increases in fat oxidation during running [23], improvements in O_2_ uptake at lactate threshold after longer-term KD [24], and even improvements in sprint performance [25]. Hence, we contend that the human body is capable of adapting to several different diets during periods of exercise training, and performance may not be compromised so long as caloric needs are met. Notwithstanding, given that KD-induced anaerobic and aerobic performance improvements were not evident herein or in other published reports, we contend that practitioners should explore implementing this diet when body composition improvements are sought rather than performance benefits.

Another interesting finding was that those in the KD group experienced increases in serum LDL cholesterol levels. Human reports are mixed with regard to how partaking in a KD affects serum LDL cholesterol levels, with some data suggesting decreases [18], no effects [20], or increases [19]. Additionally, our findings are limited in that we did not quantify LDL particle size. In this regard, Volek et al. reported that a 12-week KD intervention positively affected LDL particle size, saturated fat content in triglycerides, and Apo B/Apo A-1 ratios [18]. However, it is notable that the subjects in all of these aforementioned studies examined overweight and/or hyperlipidemic individuals. Thus, it remains plausible that otherwise healthy individuals may present increases in LDL cholesterol levels when adopting a KD. To this end, more research is needed to firmly assess how partaking in KD affects blood lipid levels in healthy versus unhealthy individuals.

## 5. Limitations and Conclusions

One obvious limitation to the current study is the limited number of participants. Hence, we feel that it is important to interpret our current data within this context. Additionally, there is the real chance that individuals in the KD group may have experienced a placebo effect in relation to performance outcomes. Alternatively stated, given that many of the KD participants experienced improvements in body composition and (anecdotally) many of these participants perceived themselves having more energy throughout the day, these phenomena could have motivated them to perform the exercise tests with more vigor relative to the CTL group.

To summarize, our data suggest that a 12-week KD in recreationally-trained CrossFit trainees facilitates improvements in whole-body adiposity without compromising weightlifting, running, or aerobic performance metrics. However, our finding that KD may reduce leg muscle mass suggests that, over prolonged periods, the diet could adversely affect muscle anabolism and warrants further human investigations using sensitive histological techniques. Additionally, given our observation that KD increased serum LDL cholesterol levels, more research is needed regarding how longer-term KD engagement affects blood lipid levels in normolipidemic athletes. Notwithstanding, the novelty of this study examining how a longer term KD affects anaerobic performance metrics continues to contribute to field of sports nutrition and challenges the paradigm regarding very high carbohydrate needs in anaerobic trainees or athletes.

## Figures and Tables

**Table 1:** Baseline characteristics between groups.

Variable	KD (*n* = 7)	CTL (*n* = 5)	*p*-Value
Males/Females (*n*)	5/2	4/1	-
Age (years)	32 ± 3	29 ± 3	0.592
Height (m)	1.71 ± 0.08	1.70 ± 0.03	0.954
Body mass (kg)	82.7 ± 8.2	76.9 ± 5.5	0.601
DXA body fat (%)	24.6 ± 2.2	20.6 ± 4.7	0.420
Strength: mass	1.35 ± 0.13	1.42 ± 0.18	0.736

Notes: Data are presented as means ± SE values. Strength: mass ratio was determined by dividing the one repetition maximum back squat (kg) by baseline body mass (kg). Abbreviations: KD, ketogenic diet group; CTL, control diet group; n, number of participants; m, meters; kg, kilograms.

**Table 2:** Changes in serum parameters between groups.

Variable	KD (*n* = 7)	CTL (*n* = 5)	Time *p*-Value Group × Time *p*-Value (η_p_^2^)
Glucose (mg/dL)			
Pre	84.4 ± 4.3	79.2 ± 5.4	0.190
Post	76.7 ± 5.5	73.0 ± 7.4	0.881 (0.002)
HDL-C (mg/dL)			
Pre	52.3 ± 3.2	56.6 ± 2.2	0.409
Post	51.3 ± 3.2	53.2 ± 3.2	0.649 (0.022)
LDL-C (mg/dL)			
Pre	114.1 ± 16.2	88.4 ± 13.9	0.147
Post	153.9 ± 27.4 *^,†^	79.0 ± 13.9 ^#^	0.029 (0.393)
Triglycerides (mg/dL)			
Pre	72.7 ± 9.5	56.8 ± 21.3	0.977
Post	65.9 ± 7.1	64.4 ± 20.7	0.585 (0.031)

Notes: Data are presented as means ± SE values. Abbreviations: KD, ketogenic diet group; CTL, control diet group; *n*, number of participants; HDL-C, high density lipoprotein cholesterol; LDL-C, low density lipoprotein cholesterol. Symbols: * increase within group from T1 to T3 (*p* < 0.05); # decrease within group from T1 to T3 (*p* < 0.05); ^†^ higher values approached significance in KD at T3 compared with CTL (*p* = 0.057).
